# Indirect CT Venography at 80 kVp with Sinogram-Affirmed Iterative Reconstruction Compared to 120 kVp with Filtered Back Projection: Assessment of Image Quality and Radiation Dose

**DOI:** 10.1371/journal.pone.0163416

**Published:** 2016-09-23

**Authors:** Inyoung Song, Jeong Geun Yi, Jeong Hee Park, Sung Min Ko

**Affiliations:** Department of Radiology, Konkuk University Medical Center, Konkuk University School of Medicine, Seoul, 143–729, Korea; University of Chicago, UNITED STATES

## Abstract

**Objective:**

To evaluate the image quality and radiation dose of indirect computed tomographic venography (CTV) using 80 kVp with sinogram-affirmed iterative reconstruction (SAFIRE) and 120 kVp with filtered back projection (FBP).

**Materials and Methods:**

This retrospective study was approved by our institution and informed consent was waived. Sixty-one consecutive patients (M: F = 27: 34, mean age 60 ± 16, mean BMI 23.6 ± 3.6 kg/m^2^) underwent pelvic and lower extremity CTVs [group A (n = 31, 120 kVp, reconstructed with FBP) vs. group B (n = 30, 80 kVp, reconstructed with SAFIRE)]. The vascular enhancement, image noise, contrast-to-noise ratio (CNR), and signal-to-noise ratio (SNR) were compared. Subjective image analysis for image quality and noise was performed by two radiologists. Radiation dose was compared between the two groups.

**Results:**

Compared with group A, higher mean vascular enhancement was observed in the group B (group A vs. B, 118.8 ± 15.7 HU vs. 178.6 ± 39.6 HU, *p* < 0.001), as well as image noise (12.0 ± 3.8 HU vs. 17.9 ± 6.1 HU, *p* < 0.001) and CNR (5.1 ± 1.9 vs. 7.6 ± 3.0, *p* < 0.001). The SNRs were not significantly different in both groups (11.2 ± 4.8 vs. 10.8 ± 3.7, *p =* 0.617). There was no significant difference in subjective image quality between the two groups (all *p* > 0.05). The subjective image noise was higher in the group B (*p* = 0.036 in reader 1, *p* = 0.005 in reader 2). The inter-observer reliability for assessing subjective image quality was good (ICC 0.746~0.784, *p* < 0.001). The mean CT dose index volume (CTDIvol) and mean dose length product (DLP) were significantly lower in group B than group A [CTDIvol, 6.4 ± 1.3 vs. 2.2 ± 2.2 mGy (*p* < 0.001); DLP, 499.1 ± 116.0 vs. 133.1 ± 45.7 mGy × cm (*p* < 0.001)].

**Conclusions:**

CTV using 80 kVp combined with SAFIRE provides lower radiation dose and improved CNR compared to CTV using 120 kVp with FBP.

## Introduction

Deep vein thrombosis (DVT) and acute pulmonary embolism (PE) are manifestations of venous thromboembolism (VTE). More than half of patients with DVT of proximal veins (iliac, femoral, and popliteal) of the pelvis and lower extremities have concurrent PE at initial presentation and more than 90% of pulmonary emboli emanate from deep veins of the legs and pelvis [1–3].

Since the combined indirect computed tomographic venography (CTV) of lower extremity and pulmonary CT angiography (CTPA) was described in 1998, this CT technique is increasingly being used and recommend for patients with suspected VTE because it can accurately depict PE to the level of segmental pulmonary arteries but also concurrently demonstrate lower extremity DVT in a single examination [4–6]. It uses only the contrast media already intravenously injected to enhance pulmonary artery and requires only additional 3 minutes to complete the overall examination [4,7,8].

However, the radiation exposure is main disadvantage of combined CTPA and CTV. With technological advances in multidetector computed tomography (MDCT), reduction of radiation dose from CT has been achieved in many ways. Among these techniques, reduction of tube voltage has been reported and shown to be effective for lower extremity CT scans [9–15]. In conjunction with low tube voltage, an iterative reconstruction (IR) algorithm which uses a correction loop in the reconstruction of an image from the raw image data to control noise on CT images has been received considerable attention in terms of maintaining image quality [16,17].

While the filtered back projection (FBP) algorithm is the conventional analytical reconstruction method and is limited in its ability to produce high quality of images at low radiation doses, the IR algorithms have been reported to allow dose reductions in the range of 30%–50% with significant noise reduction while maintaining image quality [18–20]. One of the statistical IR algorithms launched for clinical use was sinogram-affirmed iterative reconstruction (SAFIRE).

To the best of our knowledge, there are no previous studies dealing with CTV acquired with reduced tube voltage and SAFIRE. Therefore, the purpose of our study was to evaluate the objective and subjective image qualities and radiation dose of CTV at 80 kVp with SAFIRE, and to compare these with those of CTV at 120 kVp with FBP.

## Materials and Methods

### Subjects

This retrospective study was approved by the Ethics Committee and Institutional Review Board (IRB) of Konkuk University Medical Center (IRB no. KUH1140105) and informed consent was waived. Sixty-five consecutive patients underwent combined CTV and CTPA from August 2015 through March 2016 due to clinically suspected PE or DVT. Patients with severe motion or beam hardening artifacts (n = 3), inadequate vascular enhancement due to reduced cardiac ejection fraction (n = 1) were excluded. Finally, a total of 61 consecutive patients (27 male and 34 female; mean age 60 ± 16; age range 19–85 years) were included.

Among these patients, 31 patients (14 male and 17 female; mean age 62 ±16 years; age range 20–84 years) underwent CTV at 120 kVp (group A). Another 30 patients (13 male and 17 female; mean age 60 ± 17 years; age range 19–85 years) underwent CTV at 80 kVp (group B). Table 1 shows demographic findings of patients. There was no significant difference in the age (*p* = 0.759), gender distribution (*p* = 0.886), height (*p* = 0.346), body weight (*p* = 0.889), or body mass index (*p* = 0.537) between the two groups.

**Table 1:** Comparison of patient characteristics (n = 61).

Characteristics	Group A (n = 31)	Group B (n = 30)	*p* value
Age (year)	61.6 ± 15.5	60.4 ± 16.8	0.759
Gender (male/female)*	14/17	13/17	0.886
Height (cm)	161.6 ± 10.4	159.2 ± 8.2	0.346
Weight (kg)	61.1 ± 12.5	60.7 ± 10.8	0.889
BMI (kg/m^2^)	23.3 ± 3.6	23.7 ± 3.6	0.537

Group A: 120kVp and filtered back projection; Group B: 80kVp and sinogram-affirmed iterative reconstruction

Data are presented as means ± standard deviations. Differences were considered significant when *p* value was less than 0.05. Student’s *t* test and

*Mann-Whitney test were used to compare the values.

BMI body mass index

### CT protocol

All patients were examined with first-generation dual-source CT scanner (Somatom Definition, Siemens Medical Solutions, Forchheim, Germany) in the standard single-energy CT mode. An automated dual-head power injector (Stellant D, Medrad, Indianola, PA, USA) was used to administer maximum 120 mL of contrast media (Iomeron 350, iomeprol, 350 mg/ml, Bracco, Milan, Italy) at a flow rate of 4 mL/s and additional 40mL bolus of 0.9% saline solution at same flow rate through an 18-guage catheter in the antecubital vein. Automatic bolus tracking (CARE Bolus; Siemens) with a trigger attenuation threshold of 100 Hounsfield unit (HU) within the pulmonary trunk was used and acquisition of CTPA started after 5 seconds.

Acquisition of indirect CTV started 3 minutes after completion CTPA to produce near-optimum enhancement in the veins of the lower extremities [8]. CTV scanning was done in the supine position from the upper pole of the kidney to the calf in a craniocaudal direction during a single breath-hold.

In 120 kVp group, patients underwent CTV with following parameters: tube voltage, 120 kVp; collimation, 64×0.6 mm; automatic tube current modulation (ATCM, CareDose4D, Siemens) with a quality reference tube current-time product of 160 mAs; section thickness, 2.0 mm; slice interval, 2.0 mm; gantry rotation time, 0.33 seconds; and pitch, 1.4. In 80 kVp group, CT scanning was performed with same scanning parameters, except for the tube voltage of 80 kVp.

### Image reconstruction

In the 120 kVp group, raw data of CTV were reconstructed in the axial plane with a conventional FBP algorithm and a medium smooth kernel (B36f). In the 80 kVp group, images were reconstructed with a SAFIRE algorithm and the corresponding vascular kernel (I36f). With the IR algorithm, among the five adjustable strength settings (strength 1–5) which were available for adaptation of the noise model (SAFIRE), a medium strength of 3 was selected.

### Analysis of image quality

**Fig 1:**
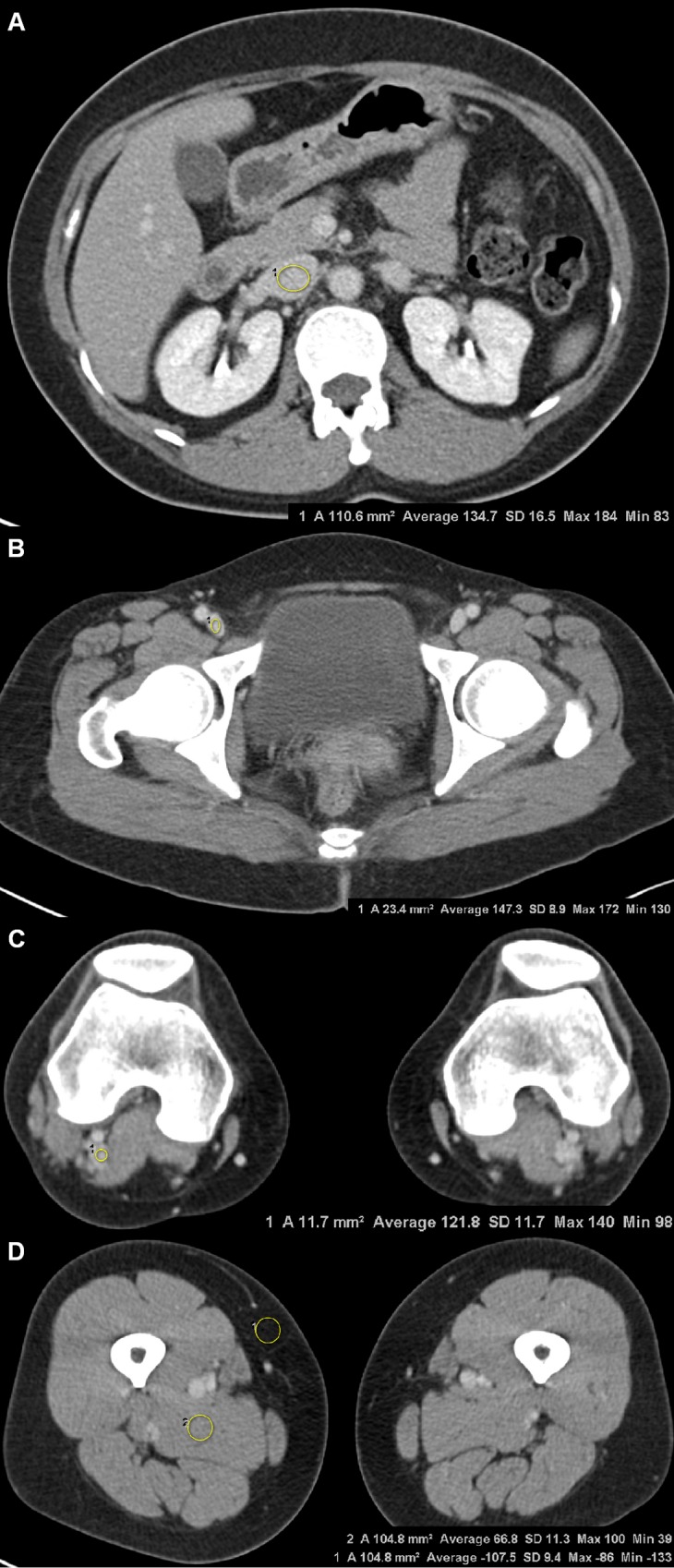
Axial CTV images obtained using 120 kVp and FBP in a 49-year-old woman with BMI 23.9. Attenuation value, vein-to-muscle contrast-to-noise ratio and signal-to-noise ratio in this patient were 134.7 HU, 7.2, and 8.2 in the IVC (a), 147.3 HU, 8.6, and 16.6 in the CFV (b), and 121.8 HU, 5.9 and 10.4 in the PV (c). Attenuation values of subcutaneous fat and muscle at the mid-thigh level were also measured using circular ROIs (d). The image quality was subjectively classified as score 4 (good) for the overall image quality and score 1 (optimal) for the image noise by the two readers. *CTV computed tomographic venography, FBP filtered back projection, BMI body mass index, HU Hounsfield unit, IVC inferior vena cava, CFV common femoral vein, PV popliteal vein, ROI region of interest.

All image data were reviewed on a Picture Archiving and Communication System (PACS, Centricity RA 1000; GE Healthcare, Barrington, IL, USA) using a window level of 100 HU and a window width of 450 HU, with manual adaptation by the reviewers if needed. All patients’ data were analyzed anonymously. For objective evaluation, a radiologist with 1 year of experience in interpreting CTV performed measurement. The vascular enhancement was quantitatively analyzed using attenuation value in HUs of following predetermined locations on axial images for each patient; the inferior vena cava (IVC) at the level of right renal vein, right common femoral vein (CFV) at the level of femoral head, and right popliteal vein (PV) at the level of popliteal fossa [11]. The mean and standard deviation (SDvein, image noise) of attenuation values of each vein were measured using a circular region of interest (ROI) that was drawn to be fitted within the vessel lumen (Fig 1). If thrombus in the right vessel was identified, the contralateral vessel was evaluated. Circular ROIs (100 ± 10 mm^2^) were also placed in homogeneous medial subcutaneous fat and muscle of mid-thigh. Contrast-to-noise ratio (CNR) and signal-to-noise ratio (SNR) were calculated using an empirically derived formula [21]; CNR = (ROIvein–ROImuscle) / SDfat, SNR = ROIvein / SDvein where the ROIvein and ROImuscle are the attenuation values of the vein and muscle, and SDfat is the noise of subcutaneous fat (standard deviation of attenuation value of subcutaneous fat).

**Fig 2:**
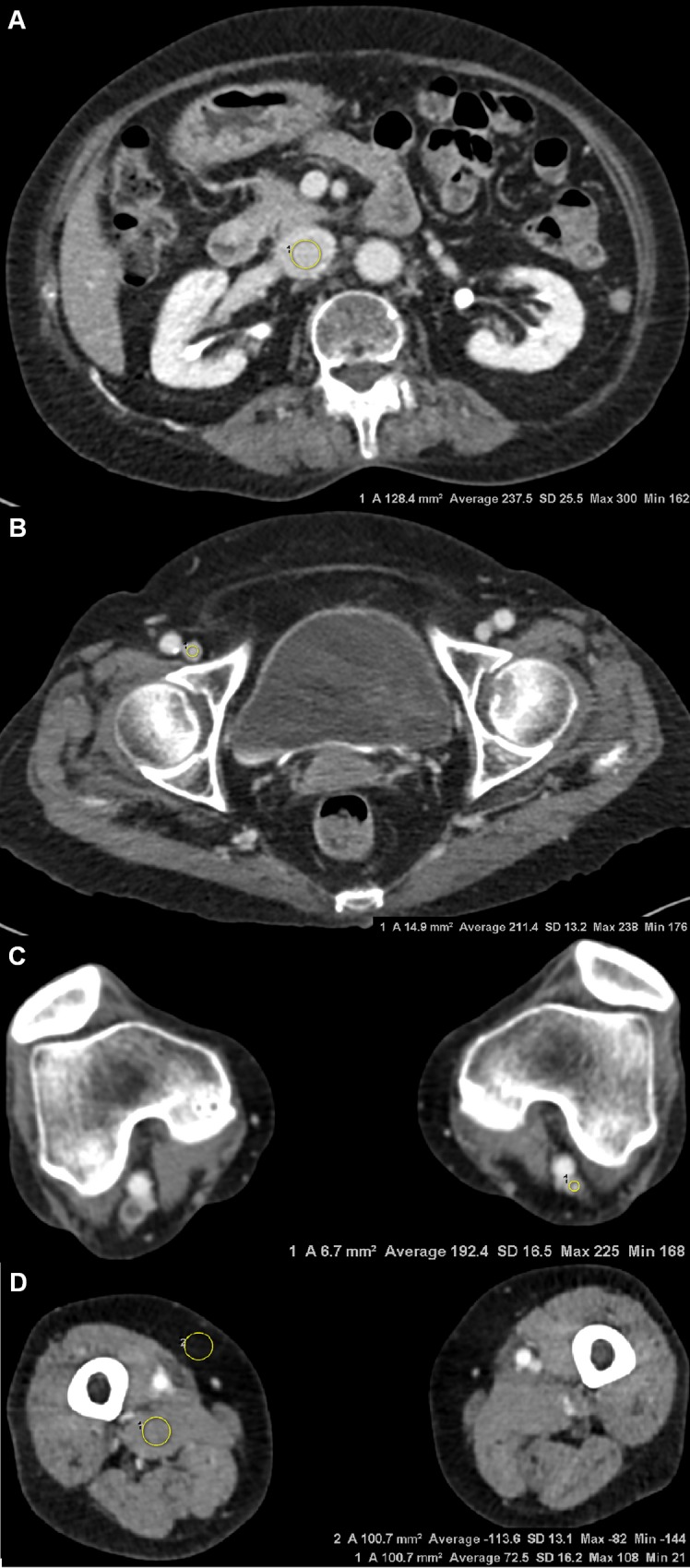
Axial CTV images obtained using 80 kVp and SAFIRE in a 75-year-old woman with BMI 23.2. Attenuation value, vein-to-muscle contrast-to-noise ratio and signal-to-noise ratio in this patient were 237.5 HU, 12.6, and 9.3 in the IVC (a), 211.4 HU, 10.6, and 16.0 in the CFV (b), and 192.4 HU, 9.2 and 11.7 in the PV (c). Attenuation values of subcutaneous fat and muscle at the mid-thigh level were also measured using circular ROIs (d). Intraluminal filling defect (arrow) was detected in the right popliteal vein, which was diagnosed as DVT. The overall image quality scores and image noise scores were 5 (excellent) and 1 (optimal) for reader 1 and 4 (good) and 1 (optimal) for reader 2. *CTV computed tomographic venography, SAFIRE sinogram-affirmed iterative reconstruction, BMI body mass index, HU Hounsfield unit, IVC inferior vena cava, CFV common femoral vein, PV popliteal vein, ROI region of interest.

For subjective analysis, two radiologists with 4 and 9 years of experience independently evaluated the randomized, axial CTV images, blinded to the scanning protocol. The readers recorded scores using a previously designed template for the following characteristics [11,22]; image quality (5, excellent, optimal enhancement to allow confident diagnosis of the presence or absence of a clot, superior to a score of 4; 4, good, optimal enhancement to allow confident diagnosis of the presence or absence of a clot; 3, equivocal, enhancement sufficient for diagnosis; 2, poor, inadequate for diagnosis of the presence or absence of a clot; 1, unacceptable, no diagnosis possible); image noise (1, optimal, none perceivable; 2, moderate, but sufficient for diagnosis; 3, unacceptable, no diagnosis possible) (Figs 1 and 2).

### Radiation dose calculation

Dose-length product (DLP) and CT dose index volume (CTDIvol) provided by the scanner system were used as CTV radiation dose descriptors.

### Statistical analysis

All statistical analyses were performed using dedicated statistical software (*PASW Statics 17*.*0*; *SPSS*, *Chicago*, *IL*, *USA*). Quantitative variables were expressed as mean ± standard deviations, while categorical variables were expressed medians with interquartile ranges (IQR). The Mann-Whitney test was used to compare categorical characteristics and the student’s *t* test was used to compare continuous variables. Intraclass correlation coefficients (ICCs) were used to assess the inter-observer reliabilities of the subjective image quality assessment. An ICC less than 0.20 indicates poor agreement; an ICC of 0.21–0.40, fair agreement; an ICC of 0.41–0.60, moderate agreement; an ICC 0.61–0.80, good agreement; and an ICC of 0.81–1.00, excellent agreement. Pearson’s correlation test was used to find out the correlation between CNR and BMI in both groups. A *p* value less than 0.05 was considered to be statistically significant.

## Results

**Fig 3:**
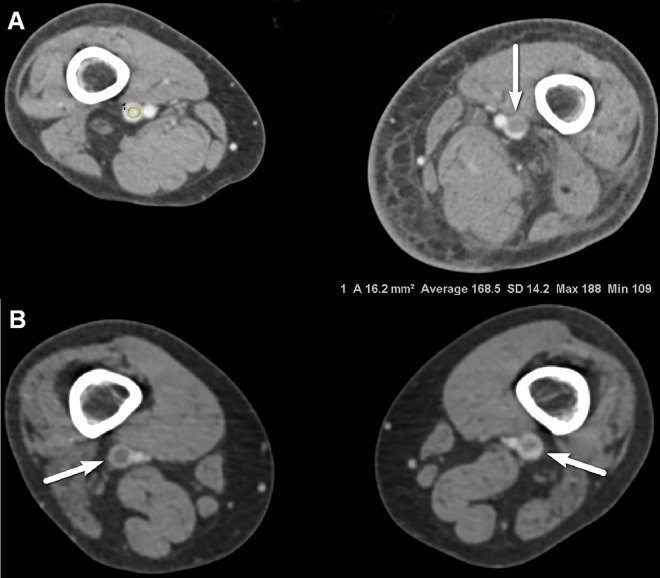
Comparison of axial CTV images obtained using (a) 120 kVp and FBP and (b) 80 kVp and SAFIRE from representative patient of each group (BMI 22.0 and 24.1, respectively). (a) Intraluminal filling defect (arrow) was detected in the left femoral vein, which was diagnosed as DVT. Attenuation value of right femoral vein, vein-to-muscle contrast-to-noise ratio and signal-to-noise ratio were 168.5 HU, 12.3 and 11.9. (b) Intraluminal filling defects (arrows) were detected in both femoral veins, which were diagnosed as DVT. Attenuation value of right common femoral vein, vein-to-muscle contrast-to-noise ratio and signal-to-noise ratio were 184.0 HU, 6.5 and 11.1 (figure not shown). Subjective scores for overall image quality and image noise were 4 (good) and 1 (optimal) in (a) and 4 (good) and 1 (optimal) in (b). *CTV computed tomographic venography, FBP filtered back projection, SAFIRE sinogram-affirmed iterative reconstruction, BMI body mass index, DVT deep vein thrombosis, HU Hounsfield unit.

Of the 61 patients, 18 had DVT (29.5%, 9 men and 9 women; mean age, 65.1 ± 13.6 years) (Fig 3). There was no significant difference in the prevalence of DVTs between the two groups [8 patients in the group A (25.8%) and 10 patients in the group B (33.3%), *p* = 0.519].

The vascular enhancement of the IVC, CFV, and PV was significantly higher in the group B than group A (*p* < 0.001) (Table 2). The mean attenuation value in the IVC was 123.0 ± 14.7 HU in the group A and 195.6 ± 33.9 HU in the group B; in the CFV, 118.2 ± 14.7 HU in the group A and 178.9 ± 37.0 HU in the group B; and in the PV, 115.1 ± 17.0 HU in the group A and 160.7 ± 41.0 HU in the group B.

**Table 2:** Results of the objective analysis of two different CTV protocols.

Parameters	ROI	Protocol		*p* value
		Group A	Group B	
Vascular enhancement (HU)	IVC	123.0 ± 14.7	195.6 ± 33.9	< .001
	CFV	118.2 ± 14.7	178.9 ± 37.0	< .001
	PV	115.1 ± 17.0	160.7 ± 41.0	< .001
	Mean	118.8 ± 15.7	178.6 ± 39.6	< .001
Image noise (HU)	IVC	15.4 ± 2.8	23.3 ± 3.1	< .001
	CFV	11.2 ± 2.2	18.6 ± 4.8	< .001
	PV	9.3 ± 3.6	11.6 ± 2.7	0.039
	Mean	12.0 ± 3.8	17.9 ± 6.1	< .001
CNR	IVC	5.3 ± 2.2	8.8 ± 2.7	< .001
	CFV	5.1 ± 2.0	7.6 ± 2.9	0.004
	PV	4.8 ± 1.6	6.4 ± 3.1	0.032
	Mean	5.1 ± 1.9	7.6 ± 3.0	< .001
SNR	IVC	8.1 ± 1.7	8.7 ± 1.4	0.275
	CFV	11.0 ± 2.3	9.8 ± 2.5	0.131
	PV	14.7 ± 6.4	14.2 ± 3.9	0.814
	Mean	11.2 ± 4.8	10.8 ± 3.7	0.617

Group A: 120kVp and filtered back projection; Group B: 80kVp and sinogram-affirmed iterative reconstruction

Data are means ± standard deviations. Student’s *t* test was used to compare the values, and differences were considered significant when *p* value was less than 0.05.

CTV computed tomographic venography, ROI region of interest, HU Hounsfield units, CNR contrast-to-noise radio, SNR signal-to-noise ratio

IVC inferior vena cava, CFV common femoral vein, PV popliteal vein

The image noise determined by standard deviation of HU in all measured veins was significantly higher in the group B than group A (*p* < 0.001). The mean image noise in the IVC was 15.4 ± 2.8 in the group A and 23.3 ± 3.1 in the group B; in the CFV, 11.2 ± 2.2 HU in the group A and 18.6 ± 4.8 HU in the group B; and in the PV, 9.3 ± 3.6 HU in the group A and 11.6 ± 2.7 HU in the group B.

The CNR was significantly higher in the group B than group A. The mean CNR in the IVC was 5.3 ± 2.2 in the group A and 8.8 ± 2.7 in the group B (*p* < 0.001); in the CFV, 5.1 ± 2.0 in the group A and 7.6 ± 2.9 in the group B (*p* = 0.004); and in the PV, 4.8 ± 1.6 in the group A and 6.4 ± 3.1 in the group B (*p* = 0.032).

There was no difference in SNR between the two groups. The mean SNR in the IVC was 8.1 ± 1.7 in the group A and 8.7 ± 1.4 in the group B (*p* = 0.275); in the CFV, 11.0 ± 2.3 in the group A and 9.8 ± 2.5 in the group B (*p* = 0.131); and in the PV, 14.7 ± 6.4 in the group A and 14.2 ± 3.9 in the group B (*p* = 0.814).

The subjective image noise of the group B was significantly higher than that of group A (*p* = 0.036 for reader 1, *p* = 0.005 for reader 2) (Table 3). However, the subjective image qualities for the both groups were not significantly different (*p* = 0.369 for reader 1, *p* = 0.454 for reader 2). The inter observer reliability for the subjective evaluation of image quality between the two reviewers was good [ICC for image quality of 0.784 (*p* < 0.001) and for image noise of 0.746 (*p* < 0.001)].

**Table 3:** Results of the subjective analysis of two different CTV protocols.

Characteristics	Readers	Protocol		*p* value
		Group A	Group B	
Image quality	R1	4 (0.5)	4 (0)	0.369
	R2	4 (0.5)	4 (0)	0.454
Image noise (HU)	R1	1 (1)	2 (1)	0.036
	R2	1 (1)	2 (0)	0.005

Group A: 120kVp and filtered back projection; Group B: 80kVp and sinogram-affirmed iterative reconstruction

Data are medians (interquartile ranges). Mann-Whitney U test was used to compare the values, and differences were considered significant when *p* value was less than 0.05.

CTV computed tomographic venography, HU Hounsfield units

The mean DLP and CTDIvol in the group B were significantly lower than those in the group A (*p* < .001) (Table 4). The mean DLP was 499.1 ± 116.0 mGy × m in the group A and 133.1 ± 45.7 mGy × cm in the group B. The mean CTDIvol was 6.4 ± 1.3 mGy in the group A and 2.2 ± 2.2 mGy in the group B.

**Table 4:** Comparison of radiation dose of two different CTV protocols.

Parameters	Group A	Group B	*p* value
DLP (mGy × cm)	499.1 ± 116.0	133.1 ± 45.7	< .001
CTDIvol (mGy)	6.4 ± 1.3	2.2 ± 2.2	< .001

Group A: 120kVp and filtered back projection; Group B: 80kVp and sinogram-affirmed iterative reconstruction

Data are means ± standard deviations. Student’s *t* test was used to compare the values, and differences were considered significant when *p* value was less than 0.05.

CTV computed tomographic venography, DLP dose-length product, CTDIvol CT dose index volume

There was no significant correlation between BMI and CNR in the group A (Pearson's correlation coefficient (ρ) = -0.239, *p* = 0.230 at the IVC; ρ = -0.078, *p* = 0.698 at the CFV; and ρ = -0.054, *p* = 0.788 at the PV) and the group B (ρ = -0.057, *p* = 0.767 at the IVC; ρ = 0.025, *p* = 0.899 at the CFV; and ρ = -0.023, *p* = 0.904 at the PV).

## Discussion

Our study showed that indirect CTV using 80 kVp combined with SAFIRE protocol (group B) yielded a significant mean increase in vascular enhancement by 50.3%, CNR by 49.0%, and comparable SNR compared with CTV using 120 kVp combined with FBP protocol (group A). Although image noise was higher in group B than group A, subjective image quality was comparable between the two groups. Furthermore, regarding the radiation dose, a significant reduction up to 73% was achieved in group B in comparison with group A when ATCM was used. To the best our knowledge, there has been no previous study about the objective/subjective image quality and radiation dose on CTV using low tube voltage setting combined with SAFIRE.

Lower tube voltage scanning is effective for radiation dose reduction because the radiation dose is approximately proportional to the square of the tube voltage [23]. Previous studies have reported that CTV using reduced tube voltage from 120 kVp to 80 kVp decreased applied radiation doses by 23% to 53% without significant degradation of diagnostic image quality [10,13–15]. In our study, mean dose reduction in CTDIvol by 73.3% and DLP by 65.6% was shown in 80 kVp protocol (group B) compared with 120 kVp protocol (group A). The reason why greater dose reduction was achieved in this study may be explained by that we used the ATCM in the angular and longitudinal directions (CARE Dose4D) while previous studies used fixed and increased tube current at 80 kVp protocol to compensate for the low tube voltage.

Lowering the tube voltage setting also results in higher intravascular enhancement given that X-ray absorption of iodine increases substantially with low effective X-ray photon energy as long as the effective photon energy remains closer to the iodine k edge of 33.2 keV [24,25]. According to the previous study by Huda et al. [25], decreasing X-ray tube voltage also decreased the average X-ray photon energy. They reported average X-ray photon energy of 43.7 keV at 80 kVp and 56.8 keV at 120 kVp. Thus, the 80 kVp CT protocol would move the average energy of X-ray spectra closer to the k edge of iodine and result in a higher mean attenuation value of iodine [26].

The higher the venous enhancement, the more the likelihood of detection of DVT would be increased. Previous studies about CTV using 120 to 150 kVp with various amounts and concentrations of contrast media have demonstrated mean venous enhancement of 95 to 118 HU [8,27,28]. In our study, the mean venous enhancement ranged 115 to 123 HU using 120 kVp and 161 to 196 HU using 80 kVp. These results are consistent with previous 80 kVp CTV studies that reported increased vascular enhancement by 19 to 53 HU compared to 120 kVp protocol [10,13,15].

In this study, no significant correlation between BMI and CNR in the both groups was observed. Given that previous studies have reported decreased image quality as the BMI being increased [29,30], our results might be due to relatively slender body size and habitus in our patients group with mean BMI of 23.6 ± 3.6 kg/m^2^ because previous results are based on patient population with BMI of 25.7 ± 4.3 kg/m^2^ in Tatsugami et al.’s study and 28.4 ± 4.1 kg/m^2^ in Brodoefel et al.’s study.

Higher image noise and reduced image quality inherent to low kVp technique have been obstacles to its widespread application in various clinical indications. The change in the image noise is known to be approximately inversely proportional to the change in the tube voltage [31,32]. In this study, the mean image noise was significantly higher in group B than A and more affected in the pelvic area. Previous studies also have demonstrated higher level of noise with lower tube voltage protocol [10,11,13–15]. However, the increased vascular enhancement with 80 kVp protocol could overcome the compromised image quality as increased vascular HU level compensated for increased level of image noise and resulted in higher CNR and comparable SNR in our study.

Furthermore, we applied SAFIRE algorithm with 80 kVp CTV and showed higher CNR and comparable SNR and subjective image quality in 80 kVp protocol compared with 120 kVp protocol. SAFIRE, one of the recently introduced IR algorithms, has been shown to be helpful to reduce image noise and improve the image quality even in the low tube voltage setting at various clinical applications [19,33–39]. Although FBP has been widely used as the conventional CT image reconstruction algorithm for decades, its drawbacks are known to be relatively high levels of image noise and streaky artifacts, particularly when low dose CT acquisition is made [22,40]. In a recent study about 70 kVp CT angiography in children, Pei et al. [36] have demonstrated that overall image quality including graininess, sharpness was significantly improved with SAFIRE, as well as a lower image noise and higher SNR and CNR were achieved with SAFIRE compared to FBP algorithm. Pontana et al. [38] have evaluated half-dose chest CT angiography reconstructed using SAFIRE and reported that SAFIRE showed similar visual perception of noise and similar overall subjective image quality and yielded significantly higher SNR and CNR compared with standard-dose FBP CT images.

Some study limitations need to be addressed. First, our study was a retrospective observation with a relatively small number of patients. Large prospective studies are required for further evaluation of the use of low tube voltage CTV using SAFIRE. Second, as we discussed prior, the BMI of our study population was relatively low compared to Western population. Third, the diagnostic accuracies of detecting DVT were not compared. However, the purpose of this study was to evaluate the radiation dose and image quality, not diagnostic accuracy. Forth, reconstruction time for IR and FBP was not compared. Because IR reconstruction requires more time than FBP, impact on clinical utility and work load should be assessed. Lastly, the potential for further contrast agent reduction with 80 kVp CTV acquisition and SAFIRE was not evaluated.

In conclusion, indirect CTV obtained at 80 kVp using the SAFIRE reconstruction provides reduced radiation dose by approximately 70% and improved CNR compared to CTV acquired at 120 kVp with FBP reconstruction. Therefore, according to our results, indirect CTV acquisition at 80 kVp with SAFIRE in patients with suspected VTE could be recommended.

## Supporting Information

S1 File Dataset for all individuals. (XLSX)
